# Nomogram based on the final antepartum ultrasound features before delivery for predicting failed spontaneous vaginal delivery in nulliparous women

**DOI:** 10.3389/fsurg.2022.1048866

**Published:** 2023-01-06

**Authors:** Ping Zhou, Han Chen, Yang Zhang, Min Yao

**Affiliations:** ^1^ Department of Gynecology, Wuhan Children's Hospital Affiliated to Tongji Medical College of Huazhong University of Science and Technology, Wuhan, China; ^2^ Department of Ultrasound, Wuhan Children's Hospital Affiliated to Tongji Medical College of Huazhong University of Science and Technology, Wuhan, China; ^3^ Department of Pediatrics, , Wuhan Children's Hospital Affiliated to Tongji Medical College of Huazhong University of Science and Technology, Wuhan, China

**Keywords:** nulliparous women, spontaneous vaginal delivery, antepartum ultrasound, nomogram, abnormal labor progression

## Abstract

**Background:**

Accurate identification of nulliparous women with failed spontaneous vaginal delivery (SVD) is crucial to minimize the hazards associated with obstetrical intervention (OI). While abnormal labor progression can be identified with intrapartum ultrasonography, labor-related complications may be unavoidable due to the limited time window left to the obstetrician. Antepartum ultrasound enables sufficient obstetric planning. However, there is typically a longer gap between ultrasound assessment and delivery that often lowers the prediction accuracy compared to intrapartum ultrasonography.

**Objective:**

In this study, antepartum ultrasound assessment was included to each fetal ultrasound examination after 36 weeks of gestation until the onset of labor. We aim to establish a nomogram to predict the likelihood of failed SVD in nulliparous women using the last antepartum ultrasound findings before labor beginning.

**Methods:**

Of the 2,143 nulliparous women recruited, 1,373 were included in a training cohort and 770 in a validation cohort, based on their delivery date. Maternal and perinatal characteristics, as well as perinatal ultrasound parameters were collected. In the training cohort, the screened correlates of SVD failure were used to develop a nomogram for determining whether a nulliparous woman would experience SVD failure. This model was validated in both training and validation cohorts.

**Results:**

SVD failure affected 217 nulliparous women (10.13%). In the training cohort, SVD failure was independently associated with BMI [odds ratio (OR) = 1.636], FHC (OR = 1.194), CL (OR = 1.398), and PCA (OR = 0.824) (all *P* < 0.05). They constituted a nomogram to estimate the individual risk of SVD failure. The model obtained clinical net benefits in both the training and validation cohorts and was validated to present strong discrimination and calibration.

**Conclusion:**

The developed nomogram based on the last antepartum ultrasound findings may be helpful in avoiding OI and its related complications by assessing the likelihood of a failed SVD in nulliparous women.

## Introduction

Nulliparous women who underwent obstetrical interventions (OI) during labor, such as operative vaginal delivery (OVD) or emergency cesarean delivery (ECD), had an approximately two-fold increased risk of postpartum hemorrhage and a nearly four-fold increased risk of neonatal intracranial hemorrhage (1–3). Therefore, accurate identification of those women with failed spontaneous vaginal delivery (SVD) is essential to minimize the hazards that come with OI.

Intrapartum ultrasound has been widely employed as a risk assessment tool for obstetricians to find abnormal labor courses in a noninvasive and objective way (4–6). With high accuracy, intrapartum ultrasound is a potentially predictive method for women who require OI due to failed SVD (7–9). However, short time windows in obstetric emergencies limit obstetric preparation (10). Labor-associated complications may be inevitable even if emergency conditions including fetal asphyxia, shoulder dystocia, and umbilical cord compression are detected by intrapartum ultrasound (4, 11).

Currently, antepartum ultrasound as an alternative option may be superior to intrapartum ultrasound in evaluating SVD failure (12, 13). It allows for making sufficient obstetric plans, thus minimizing potential delivery complications. Several studies have attempted to predict the failed SVD with antepartum ultrasound parameters, including fetal head circumference (12), cervical length (13–15), subpubic angle (12, 13), and angle of progression (16). However, these indicators seem to have lower predictive accuracy than intrapartum ultrasound (17), partially because the predictors included in their prediction models were detected in the third trimester, and the relatively large time interval between the ultrasound examination and delivery leads to unsatisfactory performance.

In the present study, antepartum ultrasound assessment was included to each fetal ultrasound examination after 36 weeks of gestation until the onset of labor. We aim to establish a nomogram to predict the likelihood of failed SVD in nulliparous women using the final antepartum ultrasound features before delivery. It will offer an intuitive and easy-to-use method for determining the likelihood of failed SVD in clinical practice. This individualized risk assessment model may be useful to avoid the adverse effects due to emergent OI.

## Materials and methods

This retrospective study was authorized by the institutional review board of Wuhan Children's Hospital, Tongji Medical College Huazhong University of Science & Technology (2021R003), and informed consents were collected from each participant. It complied with the principles stated in the Declaration of Helsinki.

### Study population

**Figure 1:**
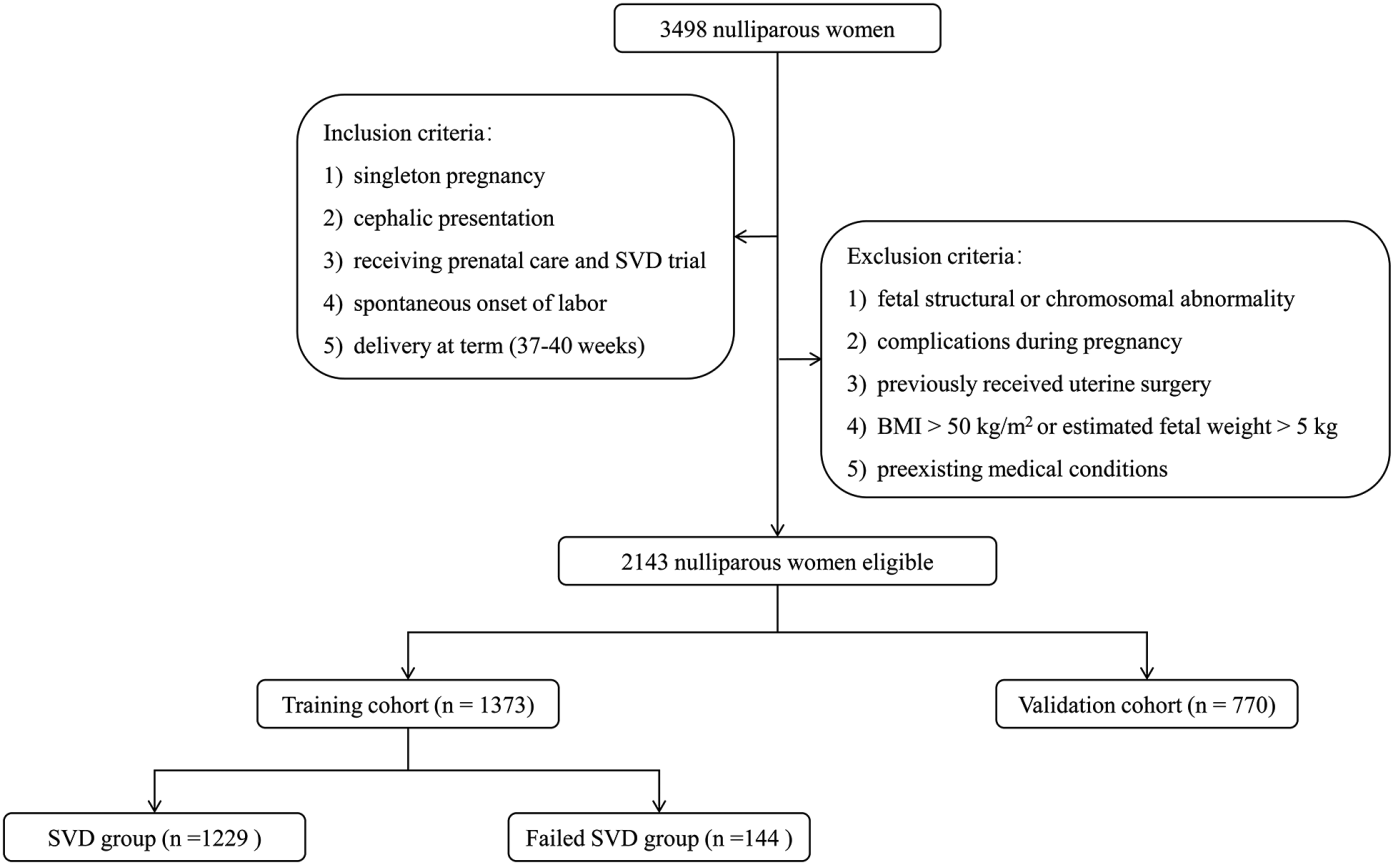
Flowchart of participant selection for building training and validation cohorts and grouping in training cohorts. BMI, body mass index; SVD, spontaneous vaginal delivery.

Between January 2019 to December 2021, the medical records of 3,498 nulliparous women in Wuhan Children's Hospital, Tongji Medical College Huazhong University of Science & Technology were reviewed for this study. Inclusion criteria were as follows: (1) singleton pregnancy, (2) cephalic presentation, (3) receiving prenatal care and SVD trial, (4) spontaneous onset of labor, and (5) delivery at term (37–40 weeks). Participants were excluded if they matched the following criteria: (1) fetal structural or chromosomal abnormality, (2) complications during pregnancy such as preeclampsia, hypertension (requiring antihypertensive medication), diabetes, renal or autoimmune disorders, (3) previously received uterine surgery such as myomectomy, (4) BMI greater than 50 kg/m^2^ or estimated fetal weight greater than 5 kg, and (5) preexisting medical conditions such as cardiac disorders, seizure disorder, or bleeding disorders. Ultimately, 2,143 nulliparous women were eligible for the analysis. To validate the established nomogram independently, women delivered from January 2019 to December 2020 were included in a training cohort (*n* = 1,373) and those from January 2021 to December 2021 were assigned to a validation cohort (*n* = 770) (Figure 1).

Maternal characteristics including age, body weight and body mass index (BMI) (obtained at around 12 weeks of gestation), gestational age at delivery, as well as delivery mode were recorded. Since 2018, antepartum ultrasound assessment for predicting delivery mode was routinely added to each fetal ultrasound examination in all nulliparous women presenting at 36 weeks of gestation until the onset of labor.

### Antepartum ultrasound assessment for delivery mode

Antepartum ultrasound assessment was performed utilizing the Voluson™ E10 (GE, Boston, MA, United States) with a C2-9 abdominal transducer and an RIC-5-9-D transvaginal transducer by two experienced sonographers.

**Figure 2:**
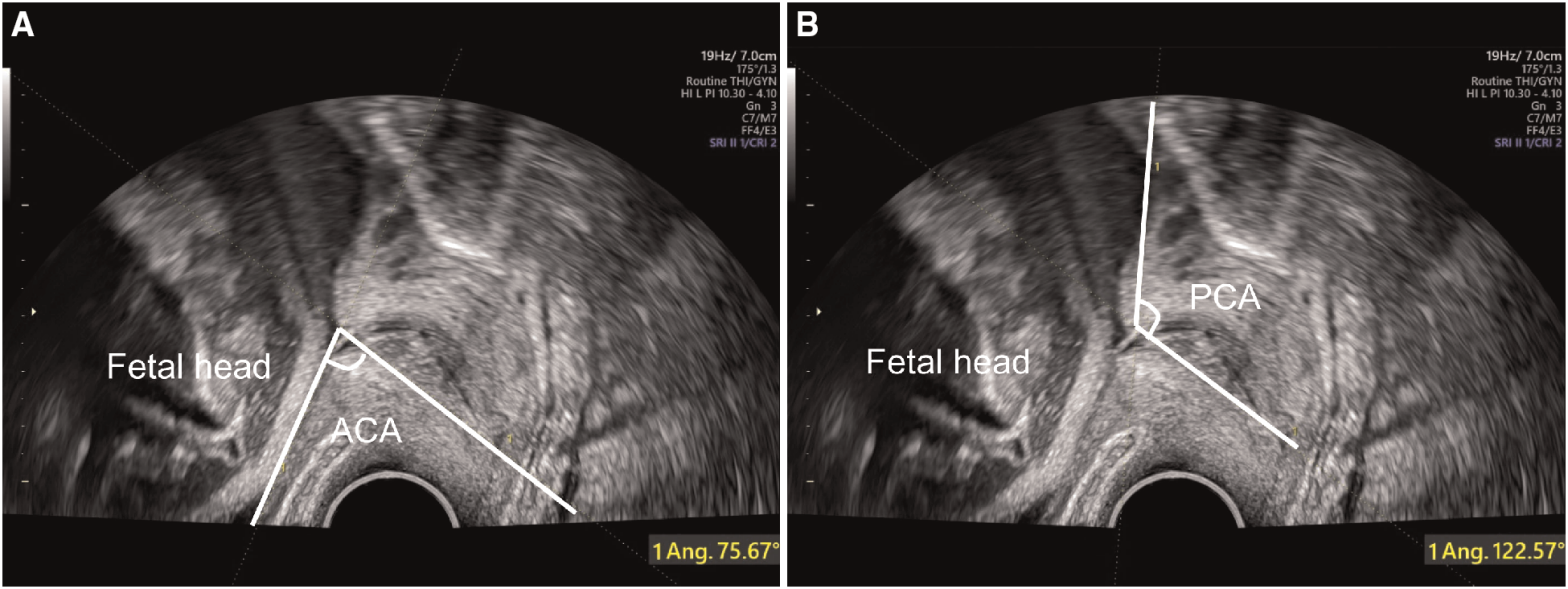
Antepartum ultrasound assessment with TVS for delivery prediction. ACA (**A**) and PCA (**B**) are assessed at the junction of the line measuring the cervical length and anterior or posterior uterine wall. TVS, transvaginal ultrasonography; ACA, anterior cervical angle; PCA, posterior cervical angle.

Fetal head circumference (FHC), abdominal circumference (AC), biparietal diameter (BPD) and femur length (FL) were measured during fetal ultrasound examination according to the guidelines of International Society of Ultrasound in Obstetrics and Gynecology (18). Estimated fetal weight (EFW) were calculated with Hadlock-4 formula (19). Following the transabdominal ultrasound, cervical length (CL), anterior cervical angle (ACA), and posterior cervical angle (PCA) were measured in the lithotomy position with transvaginal ultrasonography (TVS). CL was measured by the distance between the internal cervical orifice and the external cervical orifice, while the ACA (Figure 2A) and PCA (Figure 2B) were measured by drawing two lines that converged in the internal cervix of the orifice (20). Only the last antepartum ultrasound features before delivery were utilized for delivery prediction.

### Nomogram development

According to the SVD outcome, participants in the training cohort were assigned to successful and failed SVD groups. Despite the lack of definitive guidelines about SVD success, we determined SVD success as delivery without OVD (forceps or vacuum) and ECD, based on clinical experience and previous research (21). The association of each candidate variable with SVD failure was assessed to screen independent predictors. They were utilized to develop a nomogram for predicting the risk of failed SVD in nulliparous women. The performance of the model was first internally validated in the training cohort and then externally evaluated by fitting it to the validation cohort with the same parameter estimates.

### Statistical analysis

The Kolmogorov–Smirnov test was used to test whether continuous variables were normally distributed. They were expressed as mean ± SD and compared by independent sample t test if normally distributed; otherwise, they were expressed as median (interquartile range) and compared by Mann–Whitney *U* test. Categorical variables were compared by chi-square test. Variables with statistical significance for comparisons between groups were included in the multivariable logistic regression model, and their odds ratios (OR) as well as 95% confidence interval (CI) constituted the nomogram model.

Internal and external validations were applied to evaluate the performance of the nomogram. The discrimination of nomogram was evaluated by constructing the area under receiver operating characteristic (ROC) curve (AUC). The calibration of the nomogram was assessed by the Hosmer-Lemeshow (HL) test and displayed in the form of calibration curve. Decision curve analysis (DCA) was used to assess the clinical utility of the nomogram by quantifying the net benefit under different threshold probabilities. To decrease the overfit bias, the nomogram was validated with 1,000 bootstrap resamples.

All *p*-values were 2-sided, and the significance level was set at 0.05. SPSS version 22.0 (SPSS Inc., Chicago, IL, United States), R package version 4.1.3. and MedCalc Statistical Software version 22.0.1 (MedCalc Software Ltd, Ostend, Belgium) were used for statistical analyses.

## Result

### Participant characteristics

SVD failure affected 217 (10.13%) of 2,143 nulliparous women ultimately recruited. Most were due to prolonged labor. Among them, ECD, vacuum extraction, and forceps accounted for 74.2% (161/217), 24.0% (52/217) and 1.8% (4/217), respectively. The indications for ECD were fetal distress (52.8%, 85/161), maternal exhaustion due to prolonged duration (32.3%, 52/161), and cephalopelvic disproportion (14.9%, 24/161). Participants were divided into the training (*n* = 1,373) and validation cohorts (*n* = 770) depending on the delivery date. The comparisons of maternal characteristics and antepartum ultrasound parameters between the two cohorts are given in Table 1. There was no statistically significant difference in the incidence of failed SVD and other indicators between the two cohorts (all *p-*values > 0.05), indicating that the validation cohort might be well utilized to independently validate the prediction model.

**Table 1:** Comparisons of maternal characteristics and antepartum ultrasound parameters between the training and validation cohorts.

Indicators	Training cohort (*n* = 1,373)	Validation cohort (*n* = 770)	*t*/*U*/*χ*^2^	*p*-value
Failed SVD (*n*, %)	144 (10.48%)	73 (9.48%)	0.541^c^	0.183
Gestational age at delivery (weeks)	38 (36–39)	38 (36–39)	1.251^b^	0.211
Maternal age (years)	31.7 ± 5.9	32.0 ± 6.0	0.858^a^	0.391
Body weight (kg)	69.33 (58.43–75.76)	69.57 (59.18–76.40)	1.108^b^	0.268
BMI (kg/m^2^)	25.22 (22.62–28.28)	24.91 (21.98–28.96)	1.172^b^	0.241
FHC (mm)	345.3 ± 17.7	345.1 ± 18.0	0.855^a^	0.393
AC (mm)	273.2 ± 7.8	273.1 ± 8.3	0.393^a^	0.694
BPD (mm)	91.0 (85.2–94.3)	90.9 (86.1–96.6)	0.349^b^	0.727
EFW (g)	3344 ± 594	3305 ± 593	1.467^a^	0.142
FL (mm)	67.7 ± 4.8	68.2 ± 4.9	2.034^a^	0.062
CL (mm)	27.0 ± 3.2	26.9 ± 3.0	0.212^a^	0.833
ACA (°)	116.5 ± 24.4	117.3 ± 24.0	0.752^a^	0.452
PCA (°)	102.4 ± 29.9	102.4 ± 30.5	0.002^a^	0.998

^a^ Independent sample t-test for variables with normal distribution.

^b^ Mann–Whitney *U* test for variables with skewed distribution.

^c^ Chi-square test for categorical variables. SVD, spontaneous vaginal delivery; BMI, body mass index; FHC, fetal head circumference; AC, abdominal circumference; BPD, biparietal diameter; EFW, estimated fetal weight; FL, femur length; CL, cervical length; ACA, anterior cervical angle; PCA, posterior cervical angle.

To identify the risk factors of failed SVD, the maternal features and antepartum ultrasound parameters were compared between the successful and failed SVD groups in the training cohort (Table 2). Compared with the successful SVD group, women with higher body weight and BMI, heavier EFW, larger FHC and FL, longer CL, and narrower PCA were more likely to experience failed SVD (all *p*-values < 0.05).

**Table 2:** Perinatal characteristics and antepartum ultrasound parameters between successful and failed SVD groups.

Indicators	Successful SVD group (*n* = 1,229)	Failed SVD group (*n* = 144)	*t/U/χ* ^2^	*p*-value
Maternal age (years)	31.9 ± 5.9	32.3 ± 6.0	0.808^a^	0.419
Body weight (kg)	63.48 (58.37–70.32)	64.57 (62.28–76.14)	1.972^b^	0.049
BMI (kg/m^2^)	25.05 (22.15–27.38)	25.73 (23.26–29.33)	2.129^b^	0.033
FHC (mm)	275.4 ± 4.9	276.2 ± 10.3	6.092^a^	<0.001
AC (mm)	345.1 ± 20.3	346.3 ± 17.1	0.685^a^	0.494
BPD (mm)	91.1 (86.7–94.2)	91.5 (82.2–97.5)	0.776^b^	0.438
EFW (g)	3212 ± 494	3305 ± 590	2.123^a^	0.034
FL (mm)	67.1 ± 9.7	68.8 ± 10.1	2.033^a^	0.042
CL (mm)	27.1 ± 2.0	27.9 ± 3.6	3.953^a^	<0.001
ACA (°)	116.2 ± 24.7	119.4 ± 22.8	1.705^a^	0.088
PCA (°)	103.3 ± 28.6	99.2 ± 34.4	5.392^a^	<0.001

^a^ Independent sample *t*-test for variables with normal distribution.

^b^ Mann–Whitney *U* test for variables with skewed distribution; SVD, spontaneous vaginal delivery; BMI, body mass index; FHC, fetal head circumference; AC, abdominal circumference; BPD, biparietal diameter; EFW, estimated fetal weight; FL, femur length; CL, cervical length; ACA, anterior cervical angle; PCA, posterior cervical angle.

### Variables independently associated with SVD failure

**Figure 3:**
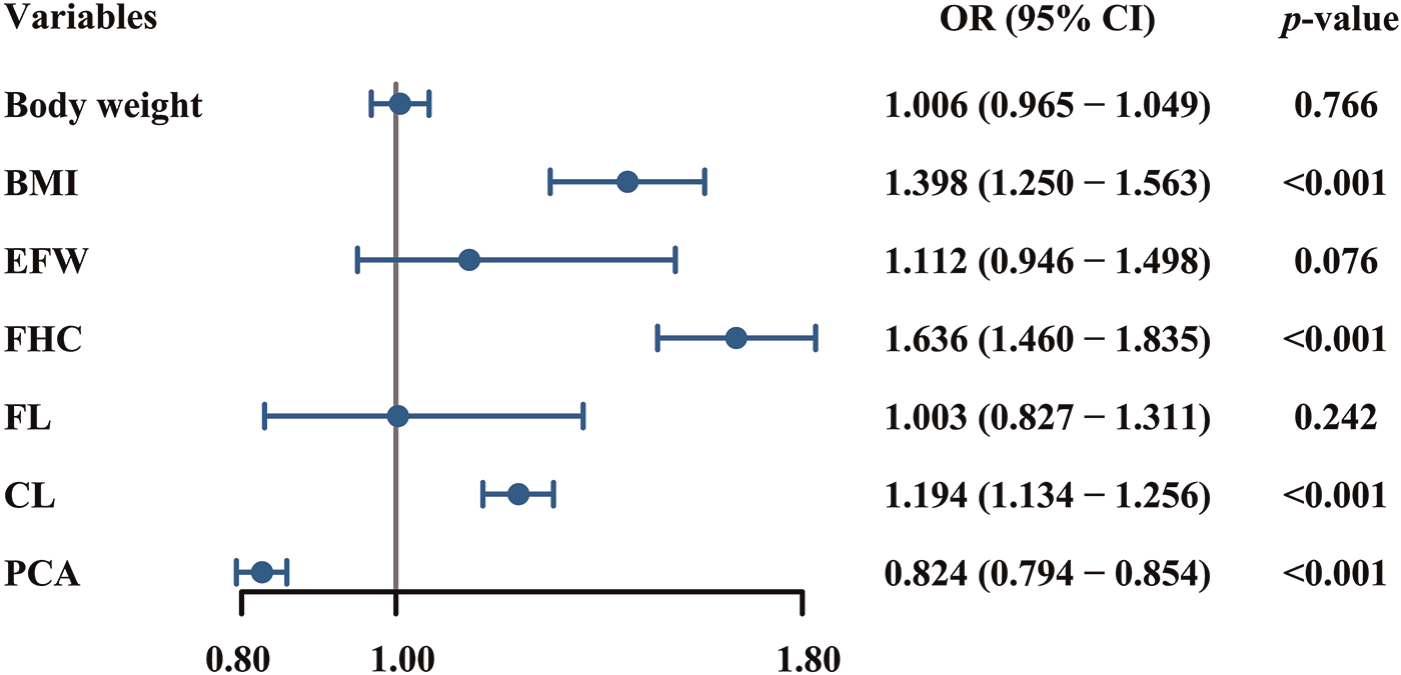
Forest plot of multivariate logistic regression analysis for the correlates of SVD failure. BMI, FHC, CL, and PCA were independently associated with SVD failure (all *p-*values <0.05). SVD, spontaneous vaginal delivery; BMI, body mass index; FHC, fetal head circumference; CL, cervical length; PCA, posterior cervical angle; OR, odds ratio.

In multivariate analysis, the variables including body weight, BMI, EWF, FHC, FL, CL, and PCA that were determined to statistically contribute to SVD failure in univariate analyses were carried over and assessed. Increased BMI (OR = 1.636, *p*-value <0.001), FHC (OR = 1.194, *p*-value <0.001), CL (OR = 1.398, *p*-value <0.001), and decreased PCA (OR = 0.824, *p*-value <0.001) were independently associated with SVD failure (Figure 3).

### Nomogram development

**Figure 4:**
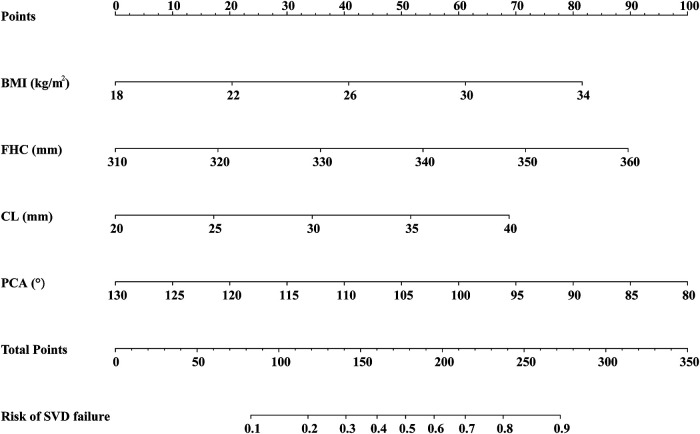
Nomogram for estimating the likelihood of SVD failure based on the multivariate logistic regression analysis. The predictors included in the nomogram are BMI, FHC, CL, and PCA. SVD, spontaneous vaginal delivery; BMI, body mass index; FHC, fetal head circumference; CL, cervical length; PCA, posterior cervical angle.

To estimate the individual risk of SVD failure, a nomogram was constructed based on BMI, FHC, CL, and PCA, which were significant predictors of failed SVD. Each variable in the nomogram was assigned a score on a point scale based on the rank order of the effect estimations. For a particular participant, the points for each predictor variable were added to get the total point, which was then used to locate the corresponding risk of SVD failure from the bottom line (Figure 4). For instance, in a nulliparous woman with a singleton pregnancy, her BMI, FHC, CL, and PCA is 22 kg/m^2^ (20 points), 350 mm (72 points), 35 mm (52 points) and 85° (90 points), respectively. With a total of almost 234 points, it was clear that the risk of SVD failure was higher than 70%, indicating that planned CD is a preferable option for this woman.

### Discrimination and calibration of the nomogram

**Figure 5:**
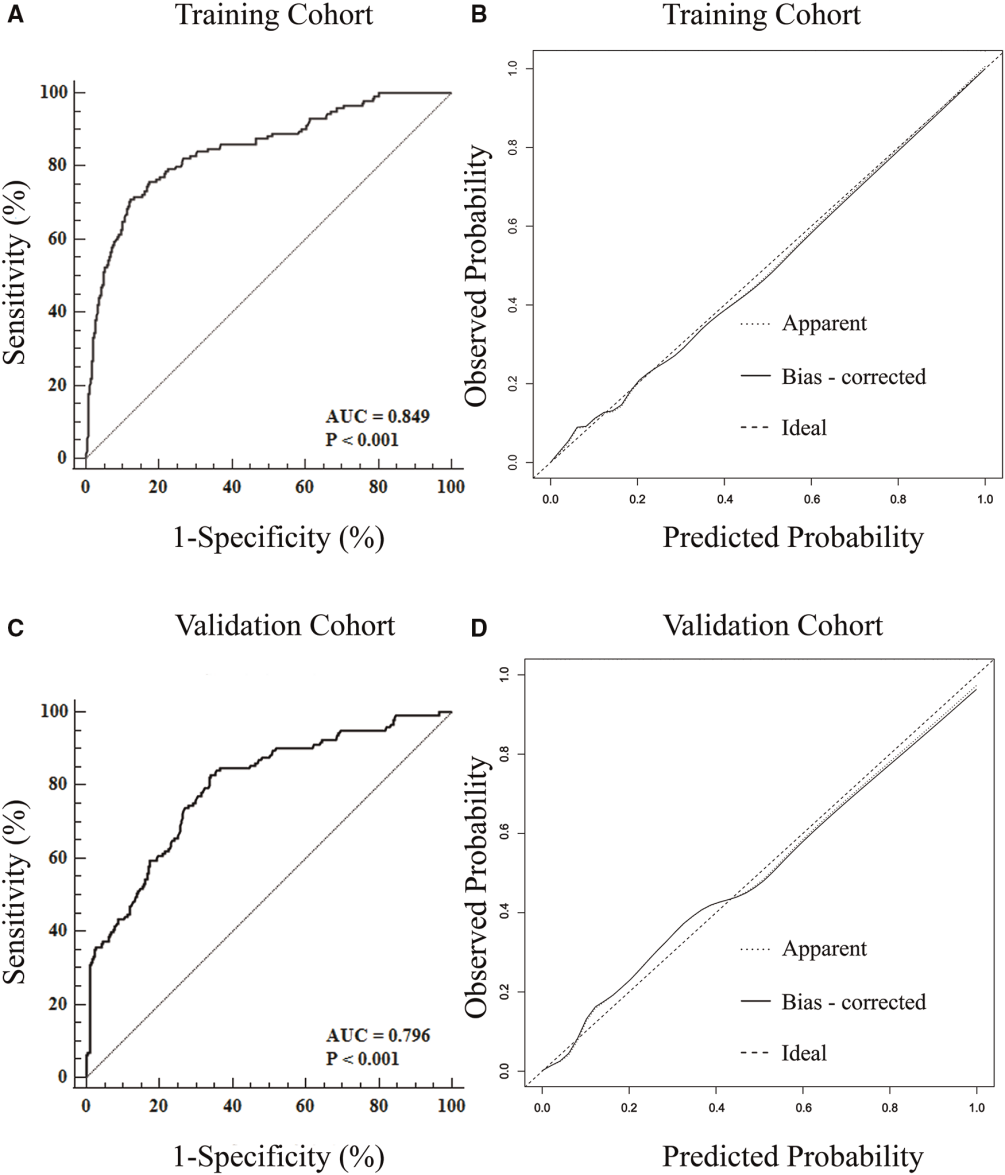
Discrimination and calibration for the nomogram in the training and validation cohorts. The AUC of ROC curves are 0.849 (**A**) and 0.796 (**C**) in the training and validation cohort, and both indicating good discrimination. The calibration curves indicate that the predicted probability of SVD failure matches the actual probability well in the training cohort (**B**) and validation cohort (**D**). SVD, spontaneous vaginal delivery; ROC, receiver operating characteristic; AUC, area under the curve.

To adjust for the bias associated with evaluating the performance of a nomogram, the assessments of discrimination and calibration were repeated for 1,000 bootstrapped samples. The accuracy of the nomogram was then further evaluated for discrimination and calibration. With an AUC of 0.849 (95% CI: 0.827–0.866), the nomogram demonstrated a strong discriminative ability of SVD failure (Figure 5A). The nomogram calibration plot indicated that the nomogram was well calibrated. The apparent line and bias-corrected line deviated only slightly from the ideal line (Figure 5B). This is further supported by the HL test (*χ*^2^ = 9.564, *p-*value = 0.364), indicating no reason to reject the null hypothesis of no difference between predicted and observed SVD failure probabilities.

The nomogram was also well evaluated in the validation cohort, with an AUC of 0.796 (95% CI: 0.773–0.817) and a non-significant *p*-value for the HL test (*χ*^2^ = 6.654, *p*-value = 0.502), again indicating good discrimination (Figure 5C) and calibration (Figure 5D).

### Clinical applicability of the nomogram

**Figure 6:**
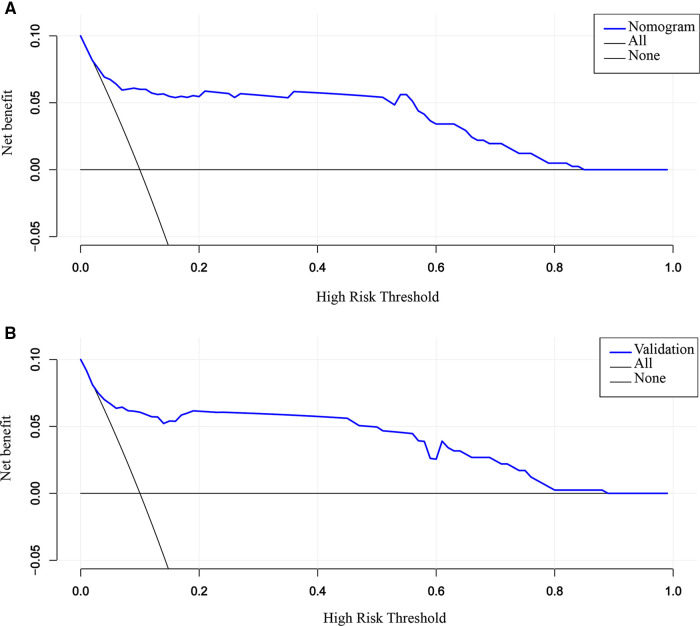
DCA for the evaluation of the clinical applicability of the nomogram. The blue line represents the net benefit. Both in the training (**A**) and validation cohort (**B**), the nomogram yields clinical net benefits when the threshold probability is between 10% and 85%. These results indicate good potential for clinical utility. DCA, decision curve analysis.

By DCA calculating the net benefit of the models across a range of thresholds for SVD failure and visualizing the results in a decision curve, the clinical usefulness of the prediction model was assessed. Specifically, if the threshold probability of a woman was between 10% and 85%, employing the nomogram to estimate the risk of SVD failure contributed greater benefit than either the “treat-all” or “treat-none” strategies (Figure 6A). The DCA plot in the validation cohort still demonstrated good net benefits, indicating that the nomogram had high potential clinical utility for nulliparous women (Figure 6B). The application of the prediction model before the labor onset will benefit the majority of nulliparous women.

## Discussion

Although intrapartum ultrasound is effective at predicting delivery mode, it only allows a limited time window for responding to emergencies during the process of labor (5, 22–24). Correspondingly, antepartum ultrasound is less accurate because it is routinely performed in the third trimester (3, 25, 26). In this study, we established a nomogram to assess the individual risk of SVD failure in nulliparous women by integrating maternal BMI and the last antepartum ultrasound findings before labor (FHC, CL, and PCA). The nomogram obtained clinical net benefits and performed well in training and validation cohorts with good discrimination and calibration. It indicated that the performance of the nomogram in predicting failed SVD was enhanced by repeated antepartum ultrasound assessment before labor. To our knowledge, this is the first nomogram for predicting the likelihood of failed SVD based on the last antepartum ultrasound features. If confirmed, it may advice nulliparous women on the most suitable mode of delivery.

Due to the constant alteration of antepartum ultrasound characteristics with advancing gestation, repeated antepartum ultrasound evaluations beyond 36 weeks allowed for a more precise detection of SVD failure than regular prenatal assessment in the third trimester (27–30). It has been noted that ultrasonic features closer to the delivery, such as the angle of progression >95° within a week of labor onset (30), predict SVD outcomes more accurately than ones farther away. Although studies have analyzed the association between the repeated evaluations and SVD outcomes, they only focus on the characteristics that lead to SVD failure and do not cover how these characteristics can be utilized to quantify the risk of SVD failure (29–31). Whereas the nomogram in the present study, which was based on the last antepartum ultrasound features, enabled a reliable evaluation during the prenatal process.

Most of the SVD failures in this study were due to prolonged labor. In order to predict the labor duration to avoid the failure before delivery, it is necessary to observe three types of indicators that reflect the force of labor, birth canal and fetus size. The developed nomogram included maternal BMI, FHC, CL, and PCA, which were revealed as the antepartum predictors of SVD failure. Obesity and larger FHC are well established to increase the risk of labor dystocia (32). Besides, TVS assessment of the cervix is considered an important tool for predicting SVD (33, 34). As crucial cervical markers, CL and PCA clearly depict the interaction between the cervical canal, internal os, and uterine wall, which is essential to the progression of labor (25, 35). The shorter the cervical length, the less resistance encountered in the descent of the fetal head (36–38). PCA is a reliable indicator of the birth canal structure. A wide PCA was associated with an easier passage of the baby through the birth canal, while a narrower PCA was more likely to cause dystocia (25). There have been reports of the use of prediction models to assess the risk of SVD failure in the obstetric population. Burke et al. (26) created a nomogram from ultrasonic data at 39 weeks and 40 weeks 6 days. However, their model might not be applicable to women who deliver at term because the majority of pregnant women give birth before 39 weeks (39). A multiparametric nomogram that included antepartum ultrasound features between 36 and 38 weeks of gestation was developed by Rizzo et al. (13). Their nomogram was inappropriate for nulliparous women because it largely focused on vaginal deliveries after CD. Unlike these previous studies, our nomogram was designed based on the features of the last antepartum ultrasound assessment in nulliparous women at term (37–40 weeks), which had broader clinical application. Obstetricians can evaluate a pregnant woman's likelihood of SVD failure if she delivers within the next week by using the risk value provided by the nomogram. If the risk is higher than 50%, more intensive monitoring is recommended. If the risk at each assessment is consistently below 50%, an SVD trial can be successfully scheduled.

There were some limitations in our study. First, we only included women delivered at 37–40 weeks to ensure the general applicability of the nomogram. But as has already been mentioned (26, 40), prolonged gestation generally appears to increase the chance of SVD failure. A prediction model that can predict the likelihood of SVD failure after 40 weeks of gestation should be developed through further study. Second, due to the retrospective study design, it was possible that some women gave delivery in other hospitals on an emergency basis during the third trimester, and the absence of this data may lead to selection bias. Third, ultrasonography is not very sensitive at advanced gestational ages, particularly in women with high BMI. Therefore, the consistency analysis of ultrasonic measurement will be necessary to improve the reproducibility in the future. Finally, even though our nomogram was validated to be reliable, the study only involved one center. Further validation with multi-center data is required to improve the model applicability.

## Conclusion

We constructed a nomogram incorporating maternal characteristics and the last antepartum ultrasound findings before delivery to assess the likelihood of failed SVD in nulliparous women. It may serve as a risk assessment tool to identify those women who are at high risk of failed SVD, thereby avoiding OI and its associated complications.

## Data Availability

The raw data supporting the conclusions of this article will be made available by the authors, without undue reservation.
